# A Systematic Review of the Inclusion of Non-Inflammatory Ultrasonographic Enthesopathy Findings in Enthesitis Scoring Indices

**DOI:** 10.3390/diagnostics11040669

**Published:** 2021-04-08

**Authors:** Sheryl Mascarenhas, Nina Couette

**Affiliations:** Department of Internal Medicine, Division of Rheumatology, The Ohio State University Wexner Medical Center, 543 Taylor Ave, Columbus, OH 43203, USA; Nina.couette@osumc.edu

**Keywords:** enthesitis, enthesopathy, spondyloarthropathy, ultrasound

## Abstract

Ultrasound has advanced the diagnosis and management of patients with inflammatory rheumatic conditions. It can be used to identify and monitor enthesitis, a cardinal feature of spondyloarthropthies. Several enthesitis scoring systems utilizing ultrasound to determine entheseal involvement have been developed. These scoring systems generally rely on determining the presence or absence of erosions, tendon enlargement, power Doppler signal, or enthesophytes. This systematic review identified ultrasound scoring systems that have been utilized for evaluating enthesitis and what key components derive the score. Review of these scoring systems, however, demonstrated confounding as some of the score components including enthesophytes may be seen in non-inflammatory conditions and some components including erosions can be seen from chronic damage, but not necessarily indicate active inflammatory disease. What is furthermore limiting is that currently there is not an agreed upon term to describe non-inflammatory enthesopathies, further complicating these scoring systems. This review highlights the need for a more comprehensive ultrasound enthesopathy scoring index.

## 1. Introduction

Detection of enthesopathy includes clinical exam and imaging. Ultrasound (US) is one of the more popular imaging modalities that has demonstrated high sensitivity and lower costs in use of detection of enthesopathy [1,2,3,4,5,6]. US can be performed at the bedside, is fast, reproducible, and preferred among many practitioners [2,3,4,5,7,8,9]. US can demonstrate features of enthesitis including tendon thickening, enthesophytes, bursitis and erosions [8]. More specifically, US can show discontinuity of tendon fibers, focal hypoechoic intratendinous areas, and fluid around the tendon [9]. More specific features within the enthesitis definition including power Doppler signal and hypervascularity are also identified with ultrasound.

Despite advances in diagnostic imaging tools, the limitations in medical language have proven to be an impediment to classifying and advancing care for patients with enthesopathies. An analysis of the medical terms enthesopathy, enthesitis, and enthesis is fundamental to developing a common working language to study patients with spondyloarthropathies (SpAs). Over the last half century, the evolution of these terms has led to changing definitions and recognition of the limitations of the current nomenclature. The prefix “enthes-” is Greek in origin, derived from “enthetikos”, which translates to “introduced into the body from without” [10,11].

Enthesis: An enthesis is the location of the insertion sites of tendons and ligaments into bones, however in 1998, McGonagle, redefined what the enthesis was, reclassifying the term enthesis as a unique organ which includes the insertion, the fibrocartilage, bursa, fat pad, adjacent trabecular bone networks, and deeper fascia [12]. Commonly considered enthesis sites include the Achilles insertion onto the calcaneus, plantar fascia insertion onto the calcaneus, and quadriceps tendon insertion at the upper patellar pole, patellar ligament at the lower patellar pole and tibial tubercle, deltoid at the acromion and clavicle, flexor and extensor tendons at the phalanges, and vertebral ligaments at the spine [13,14,15,16]. However, there are numerous insertional sites. When considering that each muscle, ligament, and joint capsule has at least two insertion points, there are estimates that suggest the human body has potentially well over a thousand enthesis sites [17].

Enthesopathy: Enthesopathy is used to describe pathologies of the tendon, ligament, joint capsule insertions, or more progressively the enthesis organ [18]. Enthesopathy is a cardinal feature of spondyloarthopathies. Several papers by Niepel et al., beginning in 1966, are credited with contributing to the initial clinical definitions of enthesopathy, describing the anatomic involvement, clinical findings, radiographic findings, and histopathology [19,20,21]. Associating enthesopathy with ankylosing spondylitis (AS) first occurred at the 1970 Heberden Oration, where John Ball presented his observations that enthesopathy primarily affected AS rather than rheumatoid arthritis (RA), rendering it a hallmark of AS [22]. Evidence of enthesopathy in the pediatric population was described in in 1982 by Rosenberg et al., where the authors described a syndrome of seronegative enthesopathy and arthropathy in children [23]. The first formalized recognition by a society occurred in 1991. The European Spondyloarthropathy Study Group (ESSG) published preliminary criteria including enthesopathy at any site within the classification criteria for SpA [24].

Enthesitis: The term enthesitis falls under the umbrella of enthesopathy. In other words, enthesitis is a type of enthesopathy. The suffix “-itis” implies inflammation [25]. Thus, enthesitis describes inflammation of the enthesis. Using McGonagle’s definition of the enthesis, enthesitis would then be defined as inflammation of the tendon, ligament, and joint capsule insertion into the bone. The suffix “-osis” implies degeneration; it has been used in other musculoskeletal syntax to describe degeneration, such as tendiniosis [26]. If one followed similar language structure, degeneration of the enthesis would be termed enthesosis. However, this is where the practice of medicine and syntax diverge.

Enthesosis: The term enthesosis, first introduced by the author in 2019 [27], was noted to be absent in general literature review, Pubmed search, Google search, and in clinical practice. Essentially this is a finding in medicine without a word to describe it. This limitation has led to sometimes inappropriate application of the term enthesitis to describe non-inflammatory conditions. Additionally, the term enthesopathy may be used, which, while correct, is not as precise as a term that could further define a lack of inflammation.

The notable absence of enthesosis has far reaching implications as it prevents researchers and physicians from advancing the field of science with regard to spondyloarthropathies and enthesopathies. For example, one study or one physician may be describing inflammatory entheseal conditions while another is describing degenerative ones, and yet since they use the exact same word to describe both differing conditions they have confounded their results. Additionally, allowing the continued use of enthesitis to substitute for enthesosis, can propagate misclassification of patients as having an active SpA when in fact they do not.

Aside from the limitations in syntax, other limitations involve assessment of enthesopathic disease activity. Some of these questions include how to measure the activity and which enthesis should be examined. Efforts to better evaluate entheseal disease activity involvement have been introduced via numerous clinical indices. The first published index was the Mander/New Castle Enthesitis Index (MEI), which included 66 enthesial sites for examination [28]. This comprehensive assessment proved to be time consuming and challenging to accomplish by physical exam alone with regard to the deeper entheseal areas [29]. More recent disease activity indices aimed to streamline the ultrasound examination. Most had an assessment of less than 12 or less examination sites, including the Belgrade Ultrasound Enthesitis Score (BUSES), Glasgow Ultrasound Enthesitis Scoring System (GUESS), and Madrid Sonography Enthesitis Index (MASEI) [30,31,32].

Enthesitis in spondyloarthropathies begins with local, destructive, microscopic, inflammatory lesions that evolve towards fibrous scarring and new bone formation [8]. Many of the scoring systems are aimed to identify these pathologic findings with the aid of US. US can identify common findings in enthesitis including decreased echogenicity of the enthesis, increased dimensions of the enthesis, structural lesions (such as enthesophytes), erosions, and increased vascularity seen on Doppler examination [8,31,33,34,35].

Changes in echogenicity that can be detected by US demonstrate localized sites of pathology. Echogenicity refers to a tissue’s ability to reflect or transmit sound waves [36]. Hyperechoic structures appear white on the US machine screen, hypoechoic structures appear gray on the screen, and anechoic structures appear black on the screen [37]. Tendons may appear hypoechoic from intrasubstance tearing and mucoid degeneration [38]. Further, the dimensions of the enthesis may be increased from secondary hypertrophy resulting from tearing and degeneration [38].

Enthesophytes are mineralized or ossified scars, also commonly referred to as bone spurs [39]. On US, these appear as a step up bony prominence at the edge of normal bone contour [31]. While this is often felt to be a feature of enthesitis, it is often found in healthy and aging individuals and does not necessarily confirm inflammation [40,41]. Osteophytes and enthesophytes are often confused. Osteophytes are considered a non-inflammatory hallmark of osteoarthritis. Enthesophytes, however, may develop at an enthesis due to mechanical stress or an inflammatory condition such a spondyloarthropathy [42]. Definitions of osteophytes and enthesophytes on ultrasound also differ. Osteophytes are defined as cortical protrusions at the joint margin seen in two planes [43]. Enthesophytes are defined as a step up of bony prominence, seen in two perpendicular planes at the end of the bone contour of the enthesis [31]. Erosions can be seen on US as cortical breakage with a step down contour defect [31].

Color PD is an important feature in evaluating enthesopathies with US. Color PD detects neovascularization of the insertion of the enthesis into the cortical bone, a feature of enthesitis [32,44,45]. In 2018, the OMERACT US initiative published consensus-based definitions regarding power Doppler in spondylarthritis. Doppler signal at the enthesis is defined as signal seen at the bone insertion (<2 mm from cortical bone). This signal must be different from reflecting surface artifact or nutrition vessel signal, with or without cortical irregularities, erosions or enthesophytes [46]. Doppler signal outside the enthesis is considered >2 mm from the cortical bone, but within the body of tendon and clearly different from nutrition vessel signals [46].

In reviewing these scoring indices, we aimed to determine how many of these SpA indices grouped non-inflammatory findings (enthesophytes and erosions) with inflammatory (power Doppler and tendon thickness) findings with regard to use of US in detecting enthesopathies. A similar review aimed at evaluation of this very question among psoriatic arthritis patients was done previously [47]. This study is an updated review expanding the population to SpA patients.

## 2. Methods

A systematic review was done by searching databases of PubMed. In addition, bibliographies of review articles were searched for relevant articles. Key index words were enthesitis, enthesis, enthesopathy, spondyloarthropathy, ultrasound, and ultrasonography. Records were limited to adult and human subjects. Published data from 2017–2021 were included in this review. Articles were reviewed for presence of an enthesopathy or enthesitis scoring index which relied on US evaluation. Indexes were evaluated for their inclusion of ultrasound findings seen in enthesitis and seen in non-inflammatory enthesopathies. Indexes not utilizing ultrasound were excluded.

## 3. Results

**Figure 1:**
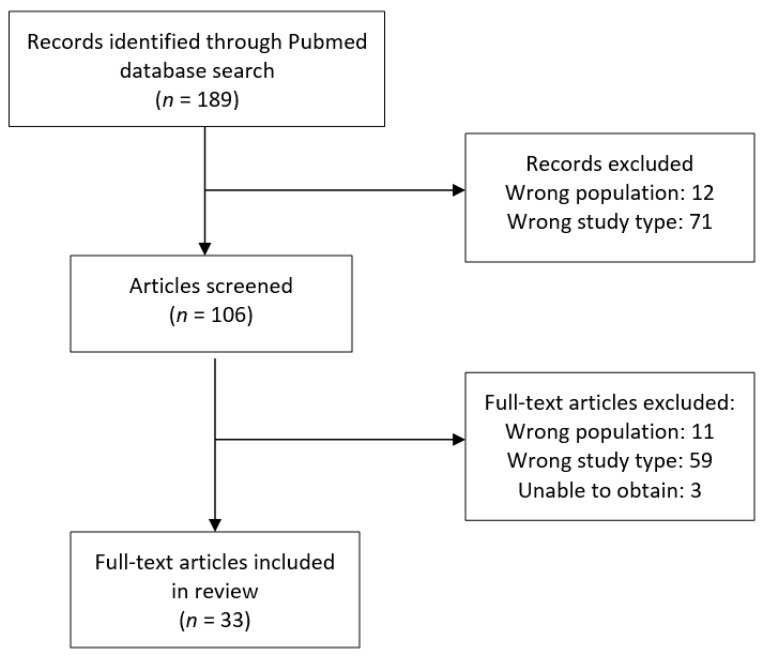
Study Design Methods.

Figure 1 demonstrates the search methods for this review. One hundred and eighty-nine records were identified in the literature search. After screening 33 articles were included in this review. Evaluation of the primary scoring index used in these studies revealed that MASEI was used in 9 studies, GUESS was used in 2 studies, the D’Agostino score was used in 2 studies, BUSES was used in 4 studies, and 11 studies used unique scoring systems. Five studies utilized multiple scoring systems including GUESS, MASEI, Spanish Enthesitis Index (SEI), and the D’Agostino score. Table 1 lists the individual index and identifies which US findings are included in scoring. Of the studies reviewed, each used a combination of inflammatory and non-inflammatory ultrasound findings to describe enthesitis.

The inclusion of power Doppler (PD) was a universal feature of all studies. Overall, 97.0% of the studies included erosions, 93.9% included enthesophytes, and 81.8% included hypoechogencity in their scoring. With regard to location, 97.0% included evaluation of the Achilles tendon, 90.9% included the patellar tendon, 75.8% included the plantar fascia, and 66.7% included the common extensor tendon of the lateral epicondyle. The number of anatomic sites used in evaluation ranged from 2 to 22, with an average of 12.7 sites.

## 4. Discussion

A previous review of PubMed and Embase databases from 1985–2010 found the most common criteria of enthesitis on US examinations included thickened entheses, hypoechogenicity, enthesophytes, bony irregularity at the enthesis, erosions, and surrounding bursitis [79]. However, others have already noted that these criteria are not actually specific for inflammation and in fact could occur instead from chronic damage and degeneration of the enthesis, highlighting the limitation of our current nomenclature and scorings systems [39].

Perhaps the most widely agreed upon definition of enthesitis as depicted by US comes from, the Outcome Measures in Rheumatology (OMERACT) US Specialist Interest Group. In 2004, OMERACT defined enthesitis on US as an “abnormally hypoechoic (loss of normal fibrillar architecture) and/or thickened tendon or ligament at its bony attachment (may occasionally contain hyperechoic foci consistent with calcification), seen in two perpendicular planes that may exhibit Doppler signal and/or bony changes, including enthesophytes, erosions, or irregularity” [80].

This definition does accurately characterize findings seen in enthesitis on US, however it does not fully separate non-inflammatory enthesopathies from inflammatory ones [81]. The only true requirement of the OMERACT definition of enthesitis is a hypoechoic tendon or ligament at the cortical attachment. While more specific inflammatory findings such as Doppler signal and erosions are modifiers within the OMERACT definition, they are not requirements. The choice of OMERACT to not require these as part of the enthesitis definition is logical as these findings may occur later on in disease course or may not be seen at all. Doppler signal, hypoechogenicity and thickening of the tendon or ligament from edema are representative of hypervascularization from active inflammatory enthesitis; However, bone erosions, calcifications, and enthesophytes reflect irreversible damage that may represent inactive disease or coexist with active inflammation [80,82]. This has limitations clinically with diagnosing and monitoring patients. It also can affect research of spondyloarthropathies, as this definition may lead to a confounding effect in study populations.

This leads to the conundrum at hand. Does one err on the side of having a broad enthesitis definition which may also include non-inflammatory conditions as well or does one err on the side of a narrow definition which may exclude some cases of enthesitis? In other words should the definition have a higher sensitivity or a higher specificity? The OMERACT definition falls more in line with the former.

Much of the previously reported literature on US use for enthesitis is based on varying scoring/classification systems, some of whose findings are not necessarily specific for inflammation but could also be found in degenerative enthesopathies and chronic microinjuries [15,83,84,85,86,87,88,89]. Our study found features of enthesophytes, tendon tearing, and tendon hypertrophy included in enthesitis scoring, despite their presence in non-inflammatory conditions. Scoring or classification systems that may include degenerative features such as enthesophytes may more accurately be classifying enthesopathies rather than specific enthesitis.

Studies have evaluated what features may be more prevalent in patients with active inflammatory conditions. Tendon thickness may be one such feature which is more specific for enthesitis and may correlate with clinical disease activity indices. Ahmed et al. compared US with the Psoriatic Arthritis Disease Activity Score (PASDAS), which is a disease activity index for psoriatic arthritis [71]. PASDAS scoring is based on a patient (PtGA) and physician (PhGA) global score, visual analog scale (VAS) score, tender (SJC66) and swollen (SJC68) joint counts, dactylitis, enthesitis, the physical component score of the short form 36 health survey (SF36-PCS), and C-reactive protein (CRP) level [90]. In the study 35 psoriatic arthritis patients were compared to 30 matched controls; the authors found Achilles tendon thickness on US in active psoriatic arthritis correlated highly with PASDAS scoring (r = 0.796, *p* < 0.001) [71].

Another distinct finding on US that may help better discern inflammatory enthesitis from an enthesosis is neovascularization. Several studies have reported the presence of blood vessels in the enthesis identified to be specific for spondyloarthropathies [14,15,31,88,89]. In one study, D’Agostino et al. evaluated entheses of 164 patients with a spondyloarthropathy, 34 with mechanical back pain (MBP) and 30 with rheumatoid arthritis (RA). The authors found a strong positive predictive value for vascularization at the enthesis, with 81% of spondyloarthropathy patients having this vascularization, but none of the patients with MBP or RA were found to have it [14].

Another study evaluating neovascularization was conducted by Poulain et al., where the sensitivity and specificity of power Doppler (PD) in identifying patients fulfilling the Assessment of SpondyloArthritis International Society (ASAS) classification criteria for axial spondyloarthropathy was investigated [44]. Those fulfilling the ASAS criteria for axial spondyloarthropathy were considered ASAS+ and those not fulfilling it were considered ASAS−. PD enthesitis was defined by the presence of vascularization at the entheseal insertion. Baseline PD was performed at eight entheseal sites on 402 patients with inflammatory back pain; PD enthesitis was detected in 58 (14.4%) patients, of which 40 (14.2%) were ASAS+ and 18 (17%) were ASAS−. The sensitivity of PD enthesitis was 13.9% and the specificity was 83.5%. The positive predictive value and negative predictive value for meeting ASAS criteria for SpA was 69% and 26.8%, respectively. Additionally, the authors found that, of the 18 ASAS− patients with positive PD, 59% fulfilled Amor’s criteria, 88% fulfilled European Spondyloarthropathy Study Group criteria, and 59% fulfilled both [44].

One could consider that the current OMERACT definition is more accurately describing enthesopathy rather than enthesitis. Further efforts are needed to better characterize a more specific definition for US findings in enthesitis. Some efforts to improve the descriptions of enthesopathy could include the use of substructure involvement and either a weighting or separate indication of specific enthesopathic findings associated more specifically with enthesitis. Another consideration is determining the most appropriate anatomic regions to include in enthesitis evaluations.

One method to improve precision of enthesopathy indices, would be for practitioners to specifically include what findings on US are visualized and what substructures are involved. Attention has been called to further describing the substructure anatomy of the enthesis. As previously reported, the enthesis organ includes collectively the insertion of the fibrocartilage, bursa, fat pad, adjacent trabecular bone networks and deeper fascia [12]. Using this terminology, the term enthesitis describes inflammation of any of these structures. While this is technically correct, it may be more precise to describe the actual substructures demonstrating pathologic features when describing enthesopathic findings [91]. This is similar to recommendations put forth by Muffuli et al. with regard to describing conditions pertaining to the hind foot and posterior ankle [92].

Use of the substructure classification would further help in describing patients with enthesopathies. For example, a patient with a retrocalcaneal bursa, Doppler signal at the enthesis and erosions would be classified as having enthesitis by most practitioners and according to most all enthesitis scoring systems. However, a patient with solely a retrocalcaneal bursitis could also be classified as having enthesitis. While this is technically correct, the two scenarios are quite dissimilar. The first is highly concerning for a spondyloarthropathy and rightly classified as an enthesitis. The second, however while could be seen in a SpA, could also be seen from mechanical changes, but justifiably still classified as an enthesitis. However, if each substructure involved were specifically defined one could more precisely and accurately describe the extent of the involvement and more finely separate patients with varying degrees of involvement rather than grouping them together.

It may be possible to further improve classification of enthesopathies by modifying scoring indices to account for the presence of inflammatory or non-inflammatory changes. For example, GUESS does not differentiate inflammatory findings from chronic damage findings in enthesopathy. A score of 1 point is offered for each finding, so for example a patient with enthesophytes that could be from chronic non-inflammatory damage may receive the same score as a patient with the same number of erosions that would be from an inflammatory condition. Having a scoring system that offers weight or at least identification of inflammatory features may offer more precision in diagnosing and monitoring enthesitis.

The development the MASEI put further emphasis on inflammatory features by adding structural aspects of the enthesis (loss of fibrillar pattern, hypoechoic aspect and fusiform thickening) and PD to their scores [32]. In addition, unlike GUESS, the MASEI is weighted. Scores can be 0 or 1 for structure, tendon thickness, and bursitis, however for erosions, calcification, and PD scores can be up to 3 [32]. This heavier weighting towards Doppler presence puts emphasis on the inflammatory findings in enthesitis. However, erosions may not necessarily indicate active inflammation and calcifications may not be feature of inflammation.

Several groups have looked at further modifications of MASEI to try to improve differentiation of inflammatory (soft tissue) findings from damage (bone) findings. Eider et al. attempted such a modification with MASEI scores. The authors developed a classification to include inflammatory changes (enthesial thickening, structural changes, bursitis and vascularization) and chronic damage changes (calcifications, enthesophytes, and erosion), which they termed MASEI-inflammatory and MASEI-damage, respectively [93]. The authors compared total MASEI scores, MASEI-inflammatory scores and MASEI-damage scores among psoriatic arthritis (PsA) patients, psoriasis patients (PsC), and healthy controls (HC). Total MASEI scores were higher in patients with PsA than PsC, with both being higher than in HC (MASEI, 13 vs. 6 vs. 3.5, respectively, *p* < 0.0001) [93]. Similarly, the MASEI-inflammatory scores were highest among the patients with PsA compared to PsC and HC (MASEI-inflammatory, 6 vs. 2 vs. 1, respectively, *p* < 0.0001) [93]. MASEI-damage scores also were higher in PsA patients compared to both patients with PsC and HC (8.5 vs. 4 vs. 3, respectively, *p* < 0.0001) [93]. Inflammatory findings were seen in all three groups; however, the highest frequency was found in patients with PsA (90%) followed by patients with PsC (72%), and was lowest in the HC (48.3%, *p* < 0.0001). The presence of power Doppler signal in at least one entheseal site was more frequent in patients with PsA compared to PsC and HC (40% vs. 18.8% vs. 8.3%, respectively, *p* < 0.0001); with no statistically significant difference between patients with PsC and HC (*p* = 0.1) [93].

Polachek et al. similarly modified MASEI, describing their divisions as a MASEI soft tissue score and bone score. The soft tissue score correlated to the definitions Eider et al. used in describing the inflammatory scores and the bone score correlated to the definitions Eider et al. used to describe damage scores [62]. In this study, Polacheck et al. compared these modified MASEI scores with radiographic findings of AS, including sacroiliitis and peripheral radiographic damage. Axial joint damage was assessed by the modified New York criteria for sacroiliitis [94] and the modified Stoke Ankylosing Spondylitis Spine Score (mSASSS) [95]. The authors reported that both the MASEI bone and soft tissue subscores were associated with higher mSASSS scores and reported an association between a higher MASEI score (10 units increase) and the presence of sacroiliitis (OR 1.33, 95% CI 1.03, 1.72) [62]. Interestingly, the MASEI bone subscore was associated with sacroiliitis, however the MASEI soft tissue subscore was not [62]. In this same study, peripheral joint damage was assessed by modified Steinbrocker score (mSS) for the hands and feet [96] and here too the authors reported both MASEI bone and soft tissue subscores were independently associated with mSS [62].

The scoring methods by both Eider et al. and Polachek et al. aim to separate out chronicity of disease. Another way to consider dividing findings is by specificity for an inflammatory condition, regardless of current disease activity. For example, The BUSES score is a weighted score, assigning more weight to the presence of power Doppler and erosions [30]. Patients with PD or erosions are assigned a score of 4 for each site of involvement, whereas increased tendon thickness, hypoechogenicity with lack of the normal fibrillary pattern, and enthesophytes are assigned a score of 1 for each site of involvement [30]. PD may signal active inflammation, whereas the erosions may signal chronic disease that may or may not have ongoing activity. This type of separation may have great merit when initially trying to diagnose a patient as this weighting will theoretically more likely identify those with more specific findings for an inflammatory spondyloarthropathy. In contrast, the system set up by Eider et al. and Polachek et al. may be helpful in monitoring for ongoing disease activity as it helps stratify active inflammatory findings from damage.

Another method to improve precision in describing enthesitis involves semi-quantification of US findings. This may be helpful in monitoring patients with regard to disease activity over time. Several groups have evaluated different methodologies for quantification and semi-quantification of various components within enthesitis indices.

D’Agostino et al. developed criteria for semi-quantification of vascularization with Doppler signal on a scale of 0–3. The vascularization was scored as 0 if Doppler signal was absent, 1 if Doppler signal was minimal (one color spot detected), 2 if Doppler signal was moderate (two spots), or 3 if Doppler signal was severe (≥three spots) [89]. This stratification is more useful in disease monitoring over time as compared to an all or none scale.

Aydin et al. modified the GUESS, offering a quantification of erosions and tendon thickness and semi-quantification of Doppler. Erosions were scored quantitatively based off the maximum diameter of the erosion. Grade 1 was an erosion diameter >0 mm but <2 mm; grade 2 was an erosion diameter ≥2 mm and <3 mm; grade 3 was an erosion diameter ≥3 mm [97]. The enthesis thickness was measured at the level of the insertions and compared to normal accepted values. Measurements that were less than 1 mm of increase exceeding the normal accepted value were scored as grade 1, 1 mm or greater but less than 2 mm of increase were scored as grade 2 and 2 mm or greater were scored as grade 3 [97]. The rest of the assessments including hypoechogencity and bursal enlargement were scored on a semi-quantitative basis with mild scored as grade 1, moderate scored as grade 2 and severe scored grade 3 [97]. For PD assessment, the authors looked for involvement in substructures of the enthesis organ [97].

Aydin et al. not only quantified and semi-quantified the findings but the authors also developed an inflammation score based off these findings. The inflammation score was composed of gray scale (GS) inflammation findings and PD scores. The GS inflammation score was calculated by adding up scores for entheseal hypoechogenicity, thickening and bursal enlargement. The Doppler inflammation score was calculated by adding up PD scores. The GS inflammation score and PD score were added to calculate the total inflammation score [97].

In this systematic review, all but one of the included scoring indices utilized the Achilles as an anatomic region of evaluation for ultrasonographic assessment of enthesitis. High resolution probes can very clearly demonstrate the Achilles, given its superficial location [7]. However, evaluation at the Achilles may be confounded by other factors such as BMI and age. Higher body mass index (BMI) and older age are associated with higher risk of developing insertional Achilles tendonopathy, with bone deformity, intratendinous calcifications, and distal tendinosis occurring more frequently in individuals with a higher BMI and older age [98,99,100]. Previous studies have reported that thickness of the Achilles tendon, and enthesophyte scores correlate with increased BMI [101,102,103]. One consideration in future development of enthesitis scoring scales would be to consider either excluding weight bearing locations or potentially having a weighted scoring system where locations that may be confounded may not score the same as locations less likely to be confounded be extrinsic factors.

Another weight-bearing enthesis that has been questioned for inclusion in scoring systems is the patellar enthesis. Overall, 90.9% of the studies in this review included the patellar tendon. Wervers et al. evaluated a modified MASEI scoring system in psoriatic arthritis patients. Their modified system excluded patellar enthesis thickness, which the authors noted was commonly found in healthy subjects, and instead added the non-weight bearing common extensor insertion at the lateral epicondyle of the elbow [61]. Interestingly, when MASEI was first introduced, the developers noted the specific exclusion of the epicondyle enthesis for concern that mechanical stress may confound the results [32]. In the Wervers et al. study, PD was scored with a semi-quantitative scoring system with no PD scored as 0, one color spot scored as 1, two color spots scored as 1.5, confluent signal scored as 2, and a severe signal scored as 3. The median MASEI score with the lateral epicondyle added was 18 (IQR 15–31) in new with PsA, 22 (IQR 15–27) in established PsA, and 10 (IQR 5–15) in healthy volunteers (*p* = 0.002) [61]. With the modifications of exclusion of the knee entheseal thickness and using the new PD scores, the modified MASEI was able to distinguish between PsA patients and healthy controls; the IQRs no longer overlapped with modified MASEI scores of 13 (IQR 10–22.5) in new PsA, 13.5 (IQR 9.5–18) in established PsA, and 3 (IQR 1–8.5) in healthy controls (*p* = 0.002) [61].

Future enthesitis scoring indices, especially in relation to psoriatic arthritis, include the nail enthesis in enthesial evaluation. The relationship of the nail and nearby extensor digitorum tendon enthesis has been recently investigated as a possible link between skin and joint disease in psoriatic arthritis [104,105]. Evaluation of the nail-enthesis with ultrasonography could be used as a potential tool to diagnose subclinical psoriatic arthritis, but the need for a standardized examination remains. Most studies done to date have been small with varying measurement techniques. Ultrasound findings including proximal nail fold thickness [106], enhanced vascularity [107] and power Doppler signal at the enthesis [108] have been suggested predictors of subclinical psoriatic arthritis. Acosta et al. showed that in subjects with clinical nail involvement there was a high proportion of extensor tendon enthesopathy detected compared to those without clinical nail involvement. This was demonstrated in patients with psoriasis and those with psoriatic arthritis [109]. Accordingly, psoriatic nail lesions can help identify the presence of enthesitis and developing thoughts on nail psoriasis indicate that it may represent entheseal disease [110]. Currently, there is no standardized ultrasound scanning technique for evaluation of nail-enthesis and a validated index could be used to help diagnose subclinical psoriatic arthritis.

## 5. Conclusions

The current nomenclature for enthesopathies is lacking. There is not an agreed upon term to describe non-inflammatory conditions of the enthesis. We propose use of enthesosis to fill this void. The limitation of not having a complete taxonomy of conditions effecting the enthesis has led to misuse of the term enthesitis. Furthermore, this is promulgated by inclusion of both inflammatory and non-inflammatory findings within currently used enthesitis scoring indices. To further advance the diagnosis, management, and research of patients with spondyloarthropathies, new working definitions need to be considered and development of US enthesitis indices with greater discernment of active inflammatory lesions are needed.

## Figures and Tables

**Table 1:** Studies Using Ultrasound to Assess Enthesitis.

Study	Up Ext	Low Ext	Sites	Hypo-Echo	Enthes-Ophytes	PD	Eros	Index	Ref.
**BUSES**									
Zuliani (2019)	CETLA	AT	12	Yes	Yes	Yes	Yes	BUSES	[48]
		PF							
		PT (P,D)							
		QT							
Hartung (2018)	CETLA CFTME	AT	14	No	No	Yes	No	BUSES	[49]
		PF							
		PT (P,D)							
		QT							
Florescu (2018)	CETLA	AT	12	Yes	Yes	Yes	Yes	BUSES	[50]
		PF							
		PT (P,D)							
		QT							
Milutonivic (2017)	CETLA	AT	12	Yes	Yes	Yes	Yes	BUSES	[51]
		PF							
		PT (P,D)							
		QT							
**D’Agostino Score**									
Yadav (2020)	CETLA CFTME	AT	10	Yes	Yes	Yes	Yes	D’Agostino	[52]
		PT							
		QT							
Zhang (2017)	CETLA	AT	12	Yes	Yes	Yes	Yes	D’Agostino	[53]
		PF							
		PT (D)							
		QT							
		GT							
**GUESS**									
Alhussain (2019)	None	AT	10	Yes	Yes	Yes	Yes	GUESS	[54]
		PF							
		PT (P,D)							
		QT							
Moshrif (2017)	None	AT	10	Yes	Yes	Yes	Yes	GUESS	[55]
		PF							
		PT (P,D)							
		QT							
**MASEI**									
Juan Molina Collada (2020)	TT	AT	12	Yes	Yes	Yes	Yes	MASEI	[56]
		PF							
		PT (P,D)							
		QT							
Vyas (2020)	TT	AT	12	Yes	Yes	Yes	Yes	MASEI	[57]
		PF							
		PT (P,D)							
		QT							
Macia-Villa (2019)	TT	AT	12	Yes	Yes	Yes	Yes	MASEI	[58]
		PF							
		PT (P,D)							
		QT							
Ishida (2019)	TT	AT	12	Yes	Yes	Yes	Yes	MASEI	[59]
		PF							
		PT (P,D)							
		QT							
Wervers (2019)	TT CETLA	AT	14	Yes	Yes	Yes	Yes	MASEI	[60]
		PF							
		PT (P,D)							
		QT							
Wervers (2018)	TT CETLA	AT	14	Yes	Yes	Yes	Yes	MASEI	[61]
		PF							
		PT (P,D)							
		QT							
Polachek (2017)	TT	AT	12	Yes	Yes	Yes	Yes	MASEI	[62]
		PF							
		PT (P,D)							
		QT							
Harman (2018)		AT	2	Yes	Yes	Yes	Yes	MASEI	[63]
Lanfranchi (2017)	TT	AT		Yes	Yes	Yes	Yes	MASEI	[64]
		PF							
		PT (P,D)							
		QT							
**Other**									
Fujikawa (2020)	CETLA	AT	14	No	Yes	Yes	Yes	Other	[65]
		PT (P,D)							
		QT							
		MCL							
		LCL							
Elnady (2019)	CETLA CFTME	AT	12	Yes	Yes	Yes	Yes	Other	[66]
		PF							
		PT (P, D)							
Macchioni (2019)	CETLA	AT	14	Yes	Yes	Yes	Yes	Other	[67]
		PF							
		PT (P,D)							
		QT							
		MCL							
Tom (2019)	CETLA	AT	22	Yes	Yes	Yes	Yes	Other	[68]
	CFTME	PF							
	TT	PT (P,D)							
	ST	QT							
	Deltoid	TPT							
Graceffa (2019)	TT CETLA	AT	16	No	Yes	Yes	Yes	Other	[69]
		PF							
		PT (P,D)							
		PL							
		MCL							
Poulain (2018)	CETLA	AT	8	No	No	Yes	No	Other	[44]
		PT (P,D)							
Balint (2018)	CETLA	AT	8	Yes	Yes	Yes	Yes	Other	[46]
		PT (P & D)							
Ruyssen-Witrand (2017)	CETLA	AT	8	No	Yes	Yes	Yes	Other	[70]
		PT (P,D)							
Ahmed (2017)	None	AT	4	No	Yes	Yes	Yes	Other	[71]
		PF							
Wink (2017)	CETLA CFTME	AT	18	Yes	Yes	Yes	Yes	Other	[72]
		PF							
		PT (P,D)							
		QT							
		PA							
		GT							
Ward (2017)	None	PTT	4	Yes	Yes	Yes	Yes	Other	[73]
		PBT							
**Multiple Scores**									
Bertolini (2020)	CETLA	AT	12	Yes	Yes	Yes	Yes	GUESS, MASEI	[74]
		PF							
		PT (P,D)							
		QT							
Martinis (2020)	CETLA	AT	12	Yes	Yes	Yes	Yes	GUESS, MASEI	[75]
		PF							
		PT (P,D)							
		QT							
Seven (2020)	CETLA TT	AT	14	Yes	Yes	Yes	Yes	GUESS, MASEI, SEI	[76]
		PT (P,D)							
		QT							
		GT							
Ozsoy-Unubol (2018)	TT CETLA CFTME	AT	18	Yes	Yes	Yes	Yes	GUESS, MASEI, D’Agostino	[77]
		PF							
		PT (P,D)							
		QT							
		TA							
Ebstein (2018)	TT CETLA	AT	14	Yes	Yes	Yes	Yes	GUESS, MASEI	[78]
		PF							
		PT (P,D)							
		QT							

Up Ext = upper extremity, Low Ext = lower extremity, Sites = number of examined sites, Hypo-echo = hypoechogenicity, PD = power Doppler, Eros = erosions, Ref = reference, CETLA = common extensor tendon of lateral epicondyle, CFTME = common flexor tendon of medial epicondyle, TT = triceps tendon, ST = supraspinatus tendon, PF = plantar fascia, PT = patellar tendon, GT = greater trochanter, TA = tibialis anterior, P = proximal, D = distal, QT = quadriceps tendon, AT = Achilles tendon, MCL = medial collateral ligament of the knee, LCL = lateral collateral ligament of the knee, PA = pes anserine, PTT = posterior tibial tendon, PBT = peroneus brevis tendon, PL = patellar ligament (tibial tuberosity), TPT = tibialis posterior tendon, BUSES = Belgrade Ultrasound Enthesitis Score, GUESS = Glasgow Ultrasound Enthesitis Scoring System, MASEI = Madrid Sonography Enthesitis Index, SEI = Spanish Enthesitis Index.

## Data Availability

The data presented in this study are available within the article and in Table 1.
